# Materia Medica and Therapeutics—Medical Chemistry—Toxicology and Legal Medicine

**Published:** 1861-03

**Authors:** Emil Fischer


### MATERIA MEDICA AND THERAPEUTICS; MEDICAL CHEMISTRY; TOXICOLOGY AND LEGAL MEDICNE.

BY EMIL FISCHER, M.D.

### MATERIA MEDICA AND THERAPEUTICS.

### I.—*On the Use of the Leaves of Globularia Alypum as a Purgative.* By ARTHUR LEARED, M.R.I.A., etc. etc.

An attempt has been lately made in France to revive the use of the leaves of Globularia Alypum as a purgative. Dr. Planchon, of Montpellier, tells us in his essay (*vide* abstract in this journal, May, 1860, p. 534,) that the qualities which recommend them are, that they neither induce nausea, vomiting or griping, that they purge without irritation, while their bitter properties give them a tonic action. Above all, that the action of the medicine is unattended by subsequent constipation; and in twenty-two detailed cases in which the drug was administered, this appears fully borne out. It was this important circumstance which induced the author to experiment with the medicine as one likely to enrich our materia medica. He prescribed the medicine for twenty out-patients at the Royal Infirmary for Diseases of the Chest, and noted the results in tabular form. A decoction of one ounce (thirty grammes, Dr. Planchon) of the leaves boiled for ten minutes in about a quarter of a pint of water, was given for a dose. Subsequently half this dose was employed, and lastly a concentrated infusion, of which half an ounce in a diluted form was the dose. With one or two exceptions, the draught was always taken before breakfast. From the table the author makes the following deductions: The intervals between taking the dose and the first evacuation, as well as the duration of action, were, as might be expected, proportionate to the amount of the dose. Thus, excluding the cases in which the periods were not noted, when one ounce of the leaves was employed, the average interval was two hours and thirty-six minutes; when half an ounce, six hours and six minutes; and when half an ounce of the infusion was used, nine hours and four minutes; and the average duration of action was respectively twenty-five hours and twenty minutes, nine hours and thirteen minutes, and five hours and five minutes. The same relation may be observed as to the number of stools, but other effects do not appear to have so completely coincided. In one case only the bowels were said to have been regulated. Constipation, varying from one to several days, occurred in thirteen cases, while the motions were more or less relaxed for one or more days in four; not mentioned in two. No griping occurred in five cases; slight griping in seven, while pain was more decided in the same number; not mentioned in one. Several of the patients were questioned as to diuretic and other effects, but nothing was elicited.

"It is plain," says Dr. Leared, "that in those cases in which the bowels were relaxed, on one or more days after the day of taking the medicine, the effect was due to a continuance of its purgative action. This happened especially after large doses as employed by Dr. Planchon, and I can only attribute the great apparent discrepancy in our results to a different interpretation of the same cause. Like other purgatives of its class, Globularia Alypum has a decidedly constipating tendency. It possesses, however, no virulent properties, but appears a safe and efficient purgative, remarkable perhaps for the duration of its action. In its griping tendencies and the nature of the stools, it is allied to senna, but from its tonic properties I assign it a position intermediate between senna and rhubarb."—(*The London Medical Review*, December, 1860.)

II.—*Means of Removing the Taste and Smell of Cod-liver Oil.* By DR. JEANNEL.

Those who have had recourse to this medicine are well acquainted with its peculiar fishy taste and smell, which to many patients are extremely disgusting. This circumstance has induced Dr. Jeannel, of Bordeaux, to make some experiments with a view to remove this inconvenience. Being aware that bitter almonds, introduced into a portion containing musk, destroy its smell immediately, and that chemists generally get rid of any smell their mortars may have contracted by rubbing them with the moist paste which remains after having prepared an emulsion of bitter almonds, it struck him that any substance containing prussic acid, like bitter almonds, might be serviceable in ridding cod-liver oil of its savor. He therefore tried the essence of bitter almonds, laurel-water, and even pure prussic acid, all with equal success. Five decigrammes of the essence will disinfect one hundred grammes of oil; in other words, it must be used in the proportion of one to two hundred. If pure prussic acid be used, the proportion is six of acid to ten thousand of oil. Laurel-water of middling strength appears to be the most convenient; the oil is shaken with once or twice its volume of laurel-water, according to the degree of infection to be removed, and the mixture then allowed to stand for forty-eight hours. When the oil is settled the laurel-water is poured off, and the oil filtered, if necessary. By this process the oil becomes quite sweet, with an agreeable taste of almonds. The oil thus prepared may be taken without any danger, even at the rate of one hundred grammes a day, and is quite as efficacious as in its natural state. Rancidity, however, cannot be got rid of in this way. Castor oil may be sweetened by a similar process; three drops of essence of bitter almonds will communicate an agreeable taste and smell to one hundred grammes of oil.—(*Chemist and Druggist*, December, 1860.)

III.—*Kousso in Tape-worm.* By M. BOUCHARDAT.

Kousso is, up to the present time, the most efficient remedy known for tænia. M. Bouchardat asserts that he has never found it fail, when properly exhibited, and he conveys to practitioners the following suggestions on the subject in the *Répertoire de Pharmacie:* An important condition for the administration of kousso, says the professor, is that, one or two days before taking the remedy, joints of tænia should have been passed. Panada is the only food allowable on the day which precedes the exhibition of the drug; half an ounce of kousso, infused in eight ounces of boiling water, is the usual dose, and should be taken cold. This draught, however, does not agree with all stomachs, and M. Bouchardat has caused M. Mentel, a dispensing chemist in Paris, to prepare, with one part of kousso and two of sugar, an ounce and a half of granules, which the patient can take fasting in five or six doses, in the space of half an hour, with a cold infusion of lime-flowers. Should thirst supervene, the patient may be allowed to drink sparingly of water. In one or two hours the bowels begin to act, and the tænia is generally passed entire, after two or three motions. Should any delay occur in the expulsion of the parasite, two ounces of castor oil in broth will secure the desired result. The tænia should be received in water at 86° Fahr.; the water is gently drained off, and, with a strong magnifier, the filiform portion of the worm should be closely inspected for the discovery of the *scolex* and hooklets.—(*Journal of Practical Medicine and Surgery*, November, 1860.)

### IV.—*Onion-juice in Ovarian Dropsy.* By M. VÉNOT.

The *Journal de Médecine* of Bordeaux contains a new case of dropsy cured by the use of onion-juice. The patient was a woman, fifty years of age, in whom three distinguished physicians of Bordeaux, MM. Vénot, Sen., Cazenave, and de Ciébra, had diagnosticated, on the 28th of January, 1860, an encysted dropsy of the right ovary of two years' duration. The consulting physicians prescribed the following remedies: Iodide of potassium in aqueous solution; baths and half-baths, with bicarbonate of soda; potions containing the same salt; mercurial frictions *larga manu;* drastics (gamboge, jalap;) then cauterization of the abdominal walls; and lastly, methodic puncturing. This rational method of treatment, which was continued for fifteen days in its medical part, was not followed by any result, and as the patient was afraid of cauteries and punctures, M. Vénot had recourse to onion-juice, as recommended by M. Serre (d'Alais) and MM. Coster and Sarraméa. The use of this strange specific was regulated as follows:—

1. Discontinuance of the medicines previously taken.
2. Abstinence from all food, and especially from ordinary drinks.
3. Half a glass of the juice of white onions, (*allium cepa vulgaris alba,*) to be taken in a cup of milk sweetened with sugar, every morning and evening.
4. During the day, two more cups of pure milk.

This treatment was stoically followed out, and soon gave encouragement of success. Toward the thirtieth day, the urine was regularly voided in great abundance, the abdomen became smaller, and after continuing the treatment for one month and a half, the patient was rid of her dropsy, and prepared herself to go to Biarritz.—(*Journal de Pharmacie et de Chimie*, December, 1860.)

### V.—*Treatment of Phthisis by Chlorate of Potassa.* By DR. E. J. FOUNTAIN.

Dr. E. J. Fountain, in the *American Medical Monthly*, communicates a paper, read by himself at the late meeting of the American Medical Association. Dr. Fountain contends that oxygen is needed in cases of phthisis, to counteract the influences resulting from imperfect aeration of the blood. He reports several cases in which the patients improved under the influence of the chlorate of potassa, of which at least half an ounce was daily administered. Dr. Fountain concludes that:—

1. The chlorate of potassa can be exhibited in large doses every day, for a long period, without injury.

2. It assists the respiratory functions by supplying the blood with oxygen.

3. It acts as a natural tonic, alterative, and depurant, by increasing the supply of that element which is the chief agent in the chemical processes in the human system.—(*New Orleans Medical News and Hospital Gazette*, December, 1860.)

### VI.—*Woorara in Epilepsy.* By M. THIERCELIN.

M. Thiercelin, struck by the counteraction of artificially-produced convulsions by woorara, has been led to administer the drug in the treatment of several convulsive diseases, more especially epilepsy, and with most marked effect. Particulars of two cases of epilepsy, which had resisted a variety of previous treatment, were laid before the members of the Academy of Sciences at their last sitting. One of the subjects treated by woorara was a young man, aged twenty-three. In him the disease was hereditary and congenital. The patient had passed four years at Charenton, and was accounted incurable. The number of attacks during the month amounted to twenty, whereof the greater part was most severe. The second case was that of a girl of seventeen, a sufferer from epilepsy for eight years past, and during the last twelve months subject to daily fits. Under the influence of the woorara treatment, (the drug being applied daily in doses varying from half a grain to a grain to the suppurating surface of a blister,) the attacks diminished in frequency so considerably that in the first case they fell in number from twenty to five per month, and in the second, from twenty-nine or thirty to eight. Not only did the frequency of the fits decrease, but a striking general improvement occurred in the health of both patients, and a marked diminution of the nervous irritability always accompanying epilepsy was also noticed. Unfortunately, the treatment could only be persisted in for eight weeks, as the stock of woorara ran short; nevertheless, the results obtained were decidedly of a nature to encourage other practitioners in following in the footsteps of M. Thiercelin.—(*Lancet*, December 1, 1860.)

### VII.—*Arsenic in Indigestion.* By M. GERMAIN.

In a paper recently read before the Academy of Medicine, by M. Germain, the author stated that remarkable success had attended his administering arsenious acid in chronic dyspepsia, and in some affections conjoined to or dependent on dyspepsia. He gives only a milligramme (one-seventieth of a grain) per diem, in the form of a pill, taken just before a meal.—(*Gaz. Hebdom.*, and *Chemist and Druggist*, December, 1860.)

### VIII.—*The Use of Ergot of Rye in Blenorrhagia, etc.* By M. LEBEL.

In a memoir upon this subject, M. Lebel states that, after a due consideration of the phenomena of contractility, which are produced by the ingestion of the ergot of rye, either medicinally or when contained in the food, he determined to avail himself of its astringent effects in affections of the canal of the urethra, of the prostate gland, and of the vagina, and that he has been very successful in curing numerous cases of mucous discharges by the administration of this drug.

Dr. Desruelles, one of the professors at Val-de-Grâce, has also for many years made use of this therapeutic agent with considerable benefit in cases of urethral discharges.—(*London Medical Review*, December, 1860.)

IX.—*Thermo-Therapeia, or the Heat Cure.* By MR. ERASMUS WILSON.

For a knowledge of thermo-therapeia, medical science is indebted to Mr. Urquhart. Thermo-therapeia is the application of atmospheric air at a high temperature to the surface of the body for the relief of pain and disease. The poets used the expression "bathed in light;" if we adopt the same language in reference to air, we may style the process a bathing in hot air, or a hot air bath; but in no other sense does the term "bath" apply to its use. I will endeavor to trace my own experience on my first introduction to the thermæ, now some time back. It was the winter time, the season bitterly cold, my inception as a "companion of the bath" took place in the private thermæ of my esteemed friend Mr. George Witt, of Prince's Terrace, Hyde Park. As an example of simplicity of construction, Mr. Witt's thermæ may be usefully taken as an illustration. He had at the back of his house a room, twenty feet long, by ten feet in breadth, and twelve feet high, with a window looking out upon a lead-flat, such as is common in London houses. To convert this room into a thermæ, he divided it into two compartments by means of a wall which crossed it about one-third from its further end. He had thus two apartments, an outer one, the cooling room or *frigidarium* of the Roman thermæ; and an inner one entered by two small doors (inner and outer) in the partition wall, the *caldarium*, *calidarium*, or *sudatorium*. Having left my garments in a portion of the outer apartment which served as a *vestiarium*, and girt about the loins with a *cummerbund*, the kilt of oriental nations, I entered the calidarium; the temperature was delicious, such a contrast with the exterior world! The wind and snow were raging without, while here was a paradise of 135 degrees of Fahrenheit. Within this hallowed nook anxiety and care and fatigue, like the burden of Bunyan's Christian, seemed to fall from my shoulders; I stretched forth my limbs in peace and enjoyment; the brain seemed to think more lightly and pleasantly, and my ideas flowed brightly and calmly.

My friend, Mr. Witt, in the course of a few minutes, was streaming with perspiration, which ran down his face in rills and dripped from his elbows and finger ends in continuous drops, while my skin was as yet dry. I was struck also with the rich and healthy complexion of his skin; it took its hues from the free circulation of the pure arterial stream through the capillary plexus of the derma; as he drew his fingers forcibly across his chest, the white traces left by their pressure were instantly replaced by the glowing vermilion of the arterial blood. There were no gorged capillaries in that skin; no venous transformation in that cutaneous plexus; no deposits of unhealthy coloring matter either in the cuticle or in the tissues beneath; no pallor, no excess and no deficiency of fat; no choked pores; no wrinkles from loss of elasticity and contractility of the fibrous and muscular structures of the corium; no abnormal or deficient sensibility of the nerves,—all was, as nature made it, perfect and beautiful. I looked for the first time in my life on a really healthy skin. How very curious and striking was the difference between my friend's skin and that of every one present; one gentleman, a finely built, handsome man, with a remarkably capacious chest, had too great a preponderance of adipose tissue, while the hue of the skin in an oblique light was a bright golden yellow. In another, the muddy tinge of the skin discovered the impure and muddy condition of the blood. The habitual use of the thermæ removes these discolorations, these indications of imperfect elimination by drainage through the perspiratory system, and while it gives beauty to the skin, bestows health on the entire economy. After a free perspiration of half an hour's duration, I was anointed with soap and had a rub down with a wisp of white fibre called *lyf*, the fibre of one of the palm trees commonly used in the East for the purpose to which it was now being applied. To the friction with soap succeeded a shower of warm water, then a douche of cold water, after which I was made to sit still for some minutes until the warmth of the skin was restored.

From the calidarium I passed therefore to the frigidarium, and on this occasion, midwinter, and a piercingly cold, snowy day, truly deserving its name. I was then *cloaked* in a sheet taken from one of the pigeon holes of the *columbarium* standing in the corner of the room, my *cummerbund* was allowed to drop on the floor, and I was made to recline upon a cane couch immediately under the open window. How cool and pleasant were the puffs of wind that played over my face and limbs—how different their impression on my skin to what they had been an hour before! I needed not the assurance of my friend that there was no fear of catarrh or bronchitis; my own feelings told me that I could resist any amount of cold, and I was obliged to suppress a longing to walk out upon the leads with no other covering than my sheet into the midst of the sleet and wind; had the lead flat been a terrace or a lawn, I could not have resisted the temptation. After awhile I exchanged the horizontal position on the couch by the open window, to a sitting posture; the sheet was thrown off from my back and limbs; the moisture of the surface was dried up, no wiping excepting of the head and face was practiced or required; the skin felt smooth and warm, and I was permitted to dress, but with the injunction that I was to dress leisurely lest the perspiration which had ceased, should again be excited. It is worthy of notice that great attention is paid to the temperature of the skin during the curriculum of the thermæ; after the cold douche, we return to the calidarium to recover any waste of heat; and in the after cooling of the body in the frigidarium, the whole of the moisture must be dried off the skin, and perspiration must be wholly suppressed, as indicated by a peculiar smoothness and polish of the surface, before we are qualified to resume our dress. All clamminess of the skin must have ceased entirely before we resort again to our usual coverings.

Among my fellow subjects for the thermæ, I have seen numerous examples of relief from painful affections dependent on morbid composition of the blood. Several were cured of gout, of rheumatism, of neuralgia. A clergyman and doctor of divinity, who resorted to the thermæ to reduce redundancy of adipose accumulation, suffered habitually during the winter season from catarrh, bronchitis, and neuralgia, and was often laid up for weeks together with these affections. Since he has adopted the use of the thermæ, which he enjoys excessively, he has diminished in bulk, he has lost all proneness to catarrh and bronchitis, and no longer experiences the pangs of neuralgia. Recently I was much interested in seeing a case of eczema of the face treated throughout by the thermal process alone; the patient lived in the thermæ for several days, he used very high temperatures, and he succeeded completely in curing his disease. It was curious, he remarked, to observe the patches of eruption; they yielded no perspiration and looked like so many parched up islets in the midst of the surrounding copiously perspiring skin. At about the same time a medical friend consulted me for prurigo senilis. "You know Mr. Witt; go and ask him to admit you to his thermæ," was my counsel. The next time I paid a visit to my friend's thermæ, there was my elderly patient luxuriating in the fullness of enjoyment. That day he left his prurigo senilis behind him in the calidarium, and I believe has had no reminder of it since. He went back to his home on the coast, and now offers a seat in his own thermæ to his curious or suffering friends.

I have hinted at the curative effects of very high temperatures; and both Mr. Urquhart and Mr. Rolland have mentioned to me important results from this process. It occurred to Mr. Urquhart's mind that as fever heat was represented by 112°, he should be able, could he create a temperature higher than fever-heat, to supersede the stage of fever at once. Thus, taking the beginning of the cold stage, which nature seemed to struggle painfully to overcome, he was enabled by a high thermal temperature to cut it short at once and to pass over it and the hot stage to that which nature seemed desirous of reaching, namely, the sweating stage. He believes that at a certain temperature he can put a stop to the fermentative process of zymotic diseases; and at a higher temperature still, destroy animal poisons. He suggests, moreover, a curious and important inquiry, namely, the influence on the chemical composition of the blood circulating through the capillary plexuses of the skin, of hot air having a temperature of 160° of Fahrenheit. Not so much its influence on the healthy blood as on the blood of persons in a state of disease.

A member of Mr. Urquhart's family, a child, was accidentally burnt; the burn was distressingly painful; various applications had been made, without relief; the child was accustomed to the thermæ, and desired to go into it; it was carried into the thermal chamber, and the pain of the burn was immediately assuaged. Mr. Urquhart himself received a severe scald; he betook himself to the thermæ; there in a heated atmosphere he directed upon the injured part a blast of air hotter than the temperature of the apartment; the pain became lessened, the process of effusion, which results in the production of a blister, was arrested; to use a popular expression, "the heat had drawn out the heat."

Looking at the thermæ in a social and political point of view, we find that it is wonderfully adapted for the preservation in health of large bodies of men, combining in itself the respective advantages of air, exercise, and ablution. Adopted by our own army, there cannot be a doubt that it would very considerably reduce the rate of sickness and death, and add to the efficiency of the men. It is applicable, also, in all cases where numbers of persons are collected together, as in barracks, prisons, poor-houses, factories, and schools; in large business establishments, where a considerable number of young men or young women are assembled; or in places of temporary meeting, as the House of Commons and clubs. It must always be borne in mind that the thermæ not only offers advantages as respects physical health, but it also conduces to moral vigor. But the usefulness of the thermæ has even a wider sphere; the Londoner, or the inhabitant of a large city, would live as healthily immured within his city walls as the rustic amid the fields and meadows of the country. His thermæ would be to him in the place of a country house, of a horse; it would give him air, exercise, freshness, health, and life.

I might add very materially to the long list of conditions to which the thermæ might be applied with advantage, but I limit myself to a single one more; it is that of extensive works, employing a large number of men, either in operations in themselves unsalutary, or in unhealthy localities. The importance of preserving a body of working men in a state of health, and in the best condition for the performance of their duties, must strike every one, and is an object worthy a moderate sacrifice on the part of proprietors or owners. There are many localities in which miasmatic fevers abound, and constantly incapacitate the working force of large operative establishments. I believe that a few pounds expended in thermæ would correct this evil; would put the men into condition to resist the miasmatic force, and to eject the poisonous elements from the blood when they had already found admission into the organism.—(*Dublin Medical Press*, October 24, 1860; abridged from the *British Medical Journal.*)

---

### Toxicology and Legal Medicine.

### I.—*Poisoning by Oxalic Acid.* By Mr. Ikin.

"A private in the left wing of the Twelfth Lancers, (recently returned from India,) a fine-looking man, twenty-six years of age, of only three or four years' service, bilious temperament, and liable at times to fits of depression, traced to anxiety about his wife and two children, he having married without leave, or rather, as was afterward ascertained, he was married when he enlisted; consequently took a false oath. He joined the regiment in India, and had only just returned, after obtaining leave of absence, from visiting his wife in London, when he committed the rash act of swallowing poison. He had just returned from duty in the riding-school, at half-past eight A.M., and had fallen from his horse, but was not injured, when he was seen mixing something in a basin at the pump, the contents of which he swallowed, dashing the basin on the ground, and remarking to a comrade near that 'he had done the service a good turn, and had taken an ounce of poison.' On returning at once to his barrack-room, he commenced to vomit and purge. He was removed immediately to the hospital, close at hand, and I was sent for. The hospital sergeant, a very intelligent man, acting under the orders of the commanding officer, who happened to be on the spot, administered an emetic, warm water, etc. I reached the hospital in twenty-five minutes from the time of his taking the poison, but he had just ceased to breathe as I entered. I continued the use of every available means for some time, but without effect. A paper, marked 'poison,' was picked up under his barrack-room window, and this contained a few grains of white powder, which I, of course, retained for analysis. The vomiting and purging continued incessantly till he died. A post-mortem examination, by order of the coroner, was made at twelve o'clock, three hours after death. The brain, heart, lungs, and kidneys were found healthy; the liver congested and slightly enlarged. The stomach and its contents were removed for analysis. On opening it, the mucous membrane was found highly congested and curiously corrugated, as if the muscular coat had been irritated and contracted. The contents of the paper were first analyzed by Mr. Reynolds and myself. On testing the white powder found in the paper, we readily detected it to be oxalic acid, and all the usual tests for the discovery of this poison gave distinct evidence of the nature of the powder The contents of the stomach were then, after the usual tedious process of boil ing and separation, put to the same tests, and gave similar results; so that we had no hesitation in pronouncing the poison taken to have been oxalic acid."—(*Lancet*, and *Dublin Medical Press*, December 26, 1860.)

II.—*Poisoning by Green Paper Hangings.*

On the ninth instant an inquest was held at Highbury, touching the death of Clarence W. King, aged three and a half years. The evidence of the father and the nurse having been given, the medical attendant of the family, J. B. Metcalf, Esq., M.R.C.S., was examined, who stated that he was called to visit the child on Thursday morning, the first instant, being informed that it was in a fit. On his arrival, the convulsions had ceased, but the little boy was in a semi-comatose state. He learned that the child had felt chilly, and had been sick; had refused his breakfast, and, therefore, had taken nothing since his supper on the previous evening. He had fallen from the sofa, two or three days previously, and there was a very slight bruise apparent on the forehead. In the evening the symptoms seemed to be somewhat relieved by the remedies administered, but during the night he became excessively restless; and another child (a little sister) being also seized with convulsions, followed by violent screaming and copious dysenteric discharge from the bowels, the medical gentleman was again sent for, and, on his arrival, found the child first affected in a state of collapse. Thinking the appearance suspicious, and the more so because, three months before, these children had been almost simultaneously attacked in a similar manner, he began to search more minutely into the causes likely to have given rise to these seizures, and discovered that they had, during the last few days, been playing with their toys in the cupboard of the breakfast-room, which room was papered with a green flock paper; that, two or three days previously, they had been amused by helping to clear out one of these cupboards, and that the little boy had sucked a piece of lace which he found among the books and toys there. These circumstances seeming to confirm his suspicions, he directed the evacuations to be preserved, and sent them to Dr. Letheby for analysis. Meanwhile every effort was made, by the application of warmth, administration of ammonia, and constant supplies of warm milk, to rally the little sufferer; but of no avail. Alternations of repose and violent tetanic convulsions continued during the day, and he died thirty-eight hours after the commencement of the attack.

A post-mortem examination was performed, and the stomach, with its contents, part of the liver, and the sigmoid flexure of the colon, were forwarded to Dr. Letheby, and at the same time, portions of the green paper.

Dr. Letheby's report was as follows: "The paper is colored with arsenite of copper, or Scheele's green, and it is so loosely attached to the surface, that it brushes off most readily. The quantity of poisonous pigment amounts to nearly one-third the weight of the paper, and, therefore, it is highly dangerous in its nature. I have examined the stomach, and find that it contains distinct evidences of arsenic; so also do the liver and the evacuations; but the quantity of poison in the last two is not nearly so large as that in the stomach."

The coroner remarked that he and a son had experienced headache from sitting in a room hung with such paper; and he having summed up, the jury returned a verdict to the following effect: "That Clarence William King had been poisoned from the inhalation of arsenical fumes, which had escaped from the green paper of a certain sitting-room, and that the manufacturer of such paper had been guilty of very careless and culpable conduct."—(*Dublin Medical Press*, November 28, 1860.)

III.—*The Antagonism of the Woorara Poison to Strychnine.* By M. Vella.

The experiments of M. Claude Bernard having demonstrated the peculiar property possessed by woorara, of paralyzing the motor nerves, the idea of employing it in tetanus, a disease of an essentially convulsive character, suggested itself to M. Vella, of Turin, and the results of very frequent experiments (nearly one hundred in number) have enabled the latter physiologist to establish his theory of the neutralization of the tetanic effects of strychnine, by the gradual injection of a solution of woorara into the veins, the jugular being the vein selected.

The experiments may be arranged into two groups. The first, in which the animals which had been poisoned by the ingestion of strychnine into the stomach, received into the blood (by injection) successive doses of woorara, from the period when the tetanic symptoms became manifest, in such a manner as to completely neutralize the toxic action of the former poison, and to perfect the re-establishment of a healthy state. In the second series of observations, M. Vella injected into the blood of certain animals a mixture of strychnine and woorara, which was attended with no ill consequences; while other animals, placed under similar conditions, died from an equivalent dose of strychnine unmixed with woorara.

Lastly, for the purpose of testing the value of his deductions, M. Vella allowed the animals, which had resisted the action of the strychnine when neutralized by the woorara, to remain at rest for some days, and then, placing them as nearly as possible under the same physiological conditions, administered, without subsequently injecting woorara, a similar dose of strychnine to that which had been employed in the previous experiments; the consequence was that these animals always rapidly succumbed to the effect of the poison.

M. Vella's observations have clearly established the fact that woorara is an antidote to strychnine, a circumstance which must be owing to the antagonism which exists between these two poisons, as a very careful examination by M Piria, a distinguished Sardinian chemist, shows that no chemical change results from their admixture.

We cannot finish this notice of M. Vella's experiments, without a reference to the laborious investigations of Dr. Harley, of University College, in connection with the action of the woorara poison in cases of tetanus and of poisoning by strychnine, which were commenced at as early a period as 1856, and which have led that observer to the same conclusions as M. Vella.—(*London Medical Review*, November, 1860.)

### IV.—*Poisoning by Cyanide of Potassium.*

A man, engaged in photography, aged thirty-six, took a draught, prepared by himself, on the 18th of December, 1858. After taking the fluid, he suddenly died. At the post-mortem examination, the external surface of the body was blanched; the death ecchymoses were well marked; the blood everywhere in the body was dark and fluid; the brain and lungs were hyperæmic. The mucous membrane of the stomach and intestines exhibited throughout no unusual appearance, (*keinen regelwidrigen Zustand.*) There were submitted, for analysis, one white medicine-glass, containing a colorless fluid, and two other glass vessels, containing the intestines. The fluid number one showed evidence of potassa and of hydrocyanic acid. The contents of the stomach consisted of a gray, dark-colored fluid, of a tolerably strong acid reaction; it had not in the least the smell of bitter almonds, but a disagreeable odor of putrefaction. The fluid parts strained off, the solid parts were found to consist of half-digested food. The fluid part was searched for cyanide of potassium. It was distilled, after addition of sulphuric acid. The fluid contained hydrocyanic acid, which was separated by the distillation; together with this acid, which was only in small quantities, there was also formic acid and fat acids.—(*Maschka, in Vierteljahrschrift*, Band ii. No 17, 1860; and *British and Foreign Med.-Chir. Review.*)

### V.—*Poisoning by Fungi.*

On the 25th of October, 1859, some fungi were served up at the breakfast of the lieutenants and ensigns in the garrison at Corte, which one of them had collected, the previous evening, in a wood of chestnut trees. Before preparing them for table, the steward had expressed his suspicions upon these fungi, but the officer insisted upon their being served up. Six officers ate of these fungi, which had preserved their natural color, and all remarked that they were *very salt.* Toward eight o'clock in the evening, ten hours after their consumption, the six officers were seized with vomiting. All believed this was due to indigestion, and they did not think of the fungi until two hours afterward. In a short time the vomiting was accompanied by colic. A medical practitioner prescribed for each of the patients an emetic draught and purgative injections. The invalids had a bad night; the vomiting and colic continued, and they experienced cramp and a burning heat at the epigastrium. Five of the officers had then recourse to various empiric remedies, which were extolled in the country. One of them, better advised, followed a rational treatment. This officer alone survived the horrible catastrophe. Four of the invalids went into the hospital three days after the poisoning. The treatment adopted especially consisted in infusions of coffee, varied frictions, mustard poultices, and purgative injections. Three sunk at the end of three days. These invalids exhibited alternations of cerebral excitement and coma. M. Chevrel has published four interesting notices on this case of poisoning, which he attributes to the spurious orange agaric, (*agaricus muscarius* of Linnæus, *amanita muscaria* of Greville.) The Army Board of Health, in consequence of this unfortunate accident, have drawn up instructions relative to edible and poisonous fungi, which we think may be profitably consulted by pharmaceutists.—(*Journal de Pharmacie*, November, 1860; and *Dublin Medical Press*, January 9, 1861.)

